# Intravenous iron sucrose v/s oral ferrous fumarate for treatment of anemia in pregnancy. A randomized controlled trial

**DOI:** 10.1186/s12884-017-1313-9

**Published:** 2017-05-08

**Authors:** Shruti B. Bhavi, Purushottam B. Jaju

**Affiliations:** 1 Gadag institute of medical sciences (GIMS), C/o Dr B R Patil, house No 6679/ B18, Near K.V.S.R College Vidyanagar extension, Gadag, Karnataka India; 2 Shri B M Patil Medical college Hospital and Research Center, B.L.D.E University’s, Bijapur, 586103 India

**Keywords:** Anemia, Iron sucrose, Ferrous fumarate, Hemoglobin, Serum ferritin

## Abstract

**Background:**

The objective of this study was to compare the efficacy, safety and tolerability of intravenous iron sucrose with that of oral ferrous fumarate in iron deficiency anemia during 14 to 34 weeks of pregnancy.

**Methods:**

A randomized controlled trial was performed involving 112 patients attending the antenatal clinic at Shri B.M.Patil Medical college Hospital, Bijapur from October 2011 to August 2012,with hemoglobin levels between 70-110 g/L and serum ferritin of < 15 ng/ml.

In the intravenous group,200 mg of iron sucrose was administered in 100 ml 0.9% sodium chloride per day. Participants in the oral group were given 200 mg of ferrous fumarate per day. The primary outcome measures for the trial, haemoglobin and serum ferritin levels were measured after 4 weeks. Statistical significance was assessed using Student’s *t*-test.

**Results:**

The change in haemoglobin in women receiving intravenous iron was higher than with oral ferrous fumarate 22 ± 11.5 g/L vs 12 ± 9 g/L (p < 0.0001).Similarly the change of serum ferritin was significantly higher in women receiving intravenous iron compared to oral iron.

55% participants in the intravenous group had an improvement in haemoglobin more than 20 g/L compared to only 11% of the oral therapy group.48% of patients in I.V group showed increase in ferritin level between 51 to 100 ng/ml in comparison to only 3.5% in oral group.

Intravenous iron sucrose is an effective in correction of anemia in pregnancy or iron store depletion.

**Conclusion:**

Intravenous iron sucrose is more effective than 200 mg a day ferrous fumarate in increasing maternal iron stores.

**Trial registration:**

The trial registration number is CTRI/2016/12/007552 registered in Clinical Trial Registry India on 8/12/2016. It is a retrospectively registered trial.

## Background

Iron deficiency anemia is the most common form of anemia the world over and also the most common nutritional disorder in the world. The overall mean global figure for the incidence of gestational anemia is 25% [1]. WHO (World Health Organisation) has estimated that prevalence of anemia in developed and developing countries in pregnant women is 14% in developed and 51% in developing countries and 65 to 75% in India [2]. It is a direct cause of 20% of maternal mortality in India [3] and indirect cause in 20 to 40% of maternal deaths [4].

Anemia interferes with the normal intrauterine growth leading to fetal loss and perinatal deaths. It is associated with increased preterm labor (28%), preeclampsia (31%) and maternal sepsis [5].

Over the past years, various oral, intramuscular and intravenous preparations of iron have been used for correction of IDA (Iron Deficiency Anemia) in pregnant patients [6]. The first choice in the treatment of iron deficiency anemia for almost all patients is oral iron replacement because of its effectiveness, safety, and lower cost [6].

The major problem with oral iron therapy in its classic ferrous form is poor tolerability and up to 40% adverse reaction rate [7]. The most common complaints are nausea, abdominal pain, diarrhea and constipation.

Severe systemic adverse effects associated with iron dextran and iron gluconate limited the use of intravenous iron. Iron sucrose complex (ISC) is a relatively new drug, which is used intravenously for the correction of IDA [8]. Iron sucrose complex is a widely used and safe molecule, which has become major interest to prevent iron deficiency anemia. The objective of this study is to compare the efficacy, safety and tolerability of intravenous iron sucrose with that of oral ferrous fumarate in iron deficiency anemia during 14 to 34 weeks of pregnancy.

## Methods

In this study, written informed consent for participation in the study was obtained from all participants.

A prospective randomized controlled study was done from October 2011 to August 2012 in the department of Obstetrics and Gynecology, Shri B M Patil Medical college of B.L.D.E University, Bijapur. 112 pregnant women between 14 to 34 weeks of pregnancy were studied. The inclusion criteria were haemoglobin level between 70 to 110 g/L, serum ferritin of less than 15 ng/ml, age 18 to 45 years, singleton pregnancy. The exclusion criteria were patients with history of bleeding tendency, history of blood transfusion within the prior 120 days, hemoglobinopathy or other red cell disorders, allergic conditions or asthma, acute inflammatory state.
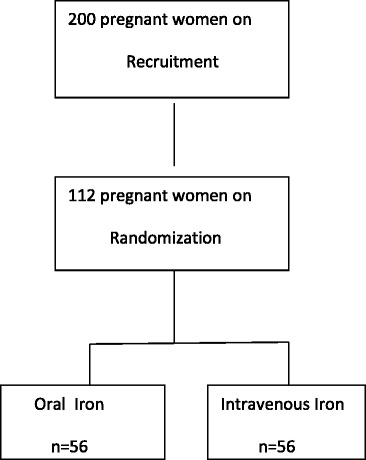


Alternatively patients were assigned to 2 groups (Group A - Oral group, Group B - Intravenous group) by simple randomization method, and 56 patients in each group were studied.

In the oral group, the patients received two tablets of ferrous fumarate, each containing 100 mg of elemental iron daily for 4 weeks.5 mg of folic acid per day was supplemented with this treatment. Patients were told to note treatment compliances carefully on a calendar provided for that purpose. Women were asked to bring back empty packs and were asked about intake of tablets and the color of the stools to ensure that they consumed the tablets.

In the intravenous group, the dose of total iron sucrose to be administered was calculated from the following formula –

Total dose required = weight in kg × (target Hb in g/L – Actual Hb in g/L) × 0.24 + 500 mg. rounded up to the nearest multiple of 100 mg [6].

This dose of iron sucrose complex was administered as 200 mg (elemental iron) in 100 ml 0.9% sodium chloride intravenously over 20 to 30 min daily up to the total dose. No test dose was given [9]. This treatment was supplemented with 5 mg of oral folic acid daily for 4 weeks to prevent an eventual folic acid deficiency and to eliminate the influence of such a deficiency on the results. Additional oral administration of iron was excluded during the 4 weeks of study.

The two groups were monitored both clinically, biologically and adverse reaction linked with it. In addition to the data required at the start of the study biologic monitoring was carried out on inclusion (day 0).

The measurements recorded were: − haemoglobin %, complete blood count, serum ferritin, urine analysis, peripheral smear for type of anemia.

After 4 weeks on day 30, haemoglobin and serum ferritin levels were repeated in both groups.

The study results were expressed as mean ± standard deviation. Further, to test the significance of difference between oral and I.V. mode of treatment in case of all parameters, student *T* test was used to verify the statistical significance.

## Results and discussion

**Fig. 1:**
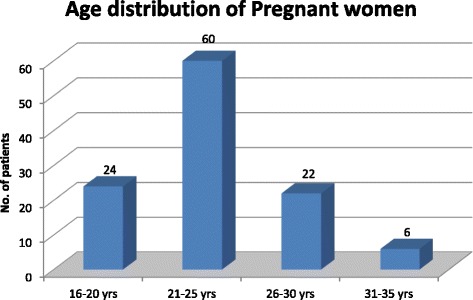
Age distribution of pregnant women

Out of 112 patients 52% of patients were between 21 to 25 years as shown in Fig. 1 and most of them were multigravida between the period 31 to 34 weeks of gestation.

Weight of women in both the groups were comparable and the difference was not statistically significant (T = 1.63, P value < 0.104).

As depicted in Table 1 a substantial increase in Hemoglobin was observed in group A (oral iron) rising from 91.4 ± 11 to 106.5 ± 10.3 g/L (Mean ± SD) as well as in group B (Intravenous iron) rising from 89 ± 10.7 to 106.4 ± 13 g/L (Mean ± SD) after 4 weeks with P value being < 0.0001 which was highly significant. Similarly there was a highly significant difference in serum ferritin levels after 4 weeks of treatment in both the groups with P value being < 0.0001 which is again highly significant as shown in Table 2.

**Table 1:** Hemoglobin level before treatment and after 4 weeks of treatment in group A (oral) and Group B (Intravenous iron)

Sl.no	Group	Parameters	Pre treatment Hb (g / L)		Post treatment Hb (g / dl)		*T*-Value	*P*-Value
			Mean	SD	Mean	SD		
1	A	Hb (g /L)	91.4	11	106.5	10.3	4.54	<0.0001
2	B	Hb (g /L)	89	10.7	106.4	13	5.62	<0.0001

**Table 2:** Serum ferritin level before treatment and after 4 weeks of treatment in group A and Group B

Sl.no	Group	Parameters	Pre treatment Serum ferritin (ng/ml)		Post treatment Serum ferritin (ng/ml)		T-Value	P-Value
			Mean	SD	Mean	SD		
1	A	S.Ferritin (ng/ml)	9.10	3.42	30.62	9.88	11.05	<0.0001
2	B	S.Ferritin (ng/ml)	8.84	3.47	120.85	87.91	7.37	<0.0001

There was no significant difference in terms of increase in hemoglobin level after 4 weeks of treatment between group A (Oral Iron) and group B (Intravenous iron) with T value 0.096 and P value being < 0.932 which is not statistically significant where as in the intravenous iron there was highly significant difference in the serum ferritin levels after 4 weeks of treatment in comparison with oral iron with T value 5.37 and P value being < 0.0001 which is highly significant as shown in Table 3.

**Table 3:** Comparison of Hemoglobin level and Serum ferritin level after 4 weeks of treatment between Group A (oral) and Group B (Intravenous iron)

	Group A		Group B		T-Value	P-Value
	Mean	SD	Mean	SD		
Post treatment –Hb (g/L)	106	10.3	106.4	13	0.096	<0.932
Post treatment-S.Ferritin (ng/ml)	30.62	9.88	120.85	87.91	5.37	<0.0001

The change in Hb % in group B (I.V group) was 22 ± 11.5 g /L (Mean ± SD) which was significantly higher in comparison with only 12 ± 9.1 g /L (Mean ± SD) in group A (Oral group) with T value 4.67 and P value being < 0.0001 which is statistically significant. The change in serum ferritin in group B (I.V group) was 112.17 ± 98.15 ng / ml (Mean ± SD) which was significantly higher in comparison with only 22.71 ± 11.32 ng/ml (Mean ± SD) in group A (Oral group) with T value 5.11 and P value being < 0.0001 which is again statistically significant.

**Fig. 2:**
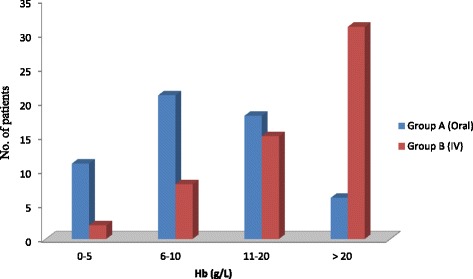
Comparison of the outcomes of treatment with oral iron versus IV iron sucrose – degree of rise in Hb g /L

A comparison of outcomes of treatment of oral iron versus I.V iron sucrose is shown in Fig. 2. 18 patients (32%) who took oral iron had an increase in Hb of 11 to 20 g/L, where as 31 patients (55%) in the IV iron sucrose group showed a greater improvement of > than 20 g/L and such a rise was seen in only 6 patients (11%) of the oral iron group. The differences in the responses were highly significant (p < 0.0001).

**Fig. 3:**
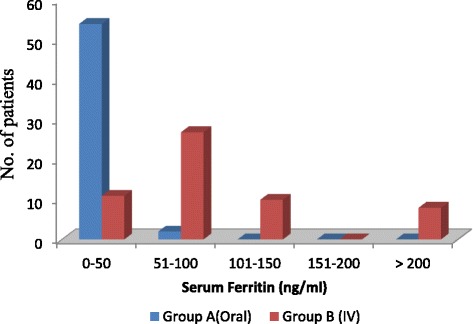
Comparison of outcomes of treatment with oral iron versus IV iron sucrose – degree of increase in Serum Ferritin (ng / ml)

As depicted in Fig. 3 out of 56 patients treated with oral iron, 54 patients (96%) showed an increase in serum ferritin levels up to 50 ng / ml and only 2 patients (3.5%) had increase in serum ferritin between 51 to 100 ng / ml whereas in the IV group 27 patients (48%) showed increase in serum ferritin levels between 51 to 100 ng / ml. 10 patients treated with I.V iron (18%) had increase in serum ferritin by 101 to 150 ng / ml and 8 patients (14%) had increase in serum ferritin by more than 200 ng / ml and none of the patients in orally treated group had any rise in serum ferritin levels > 100 ng / ml. The differences in the responses were highly significant (p < 0.0001).

In the Group B, those treated with I.V iron sucrose 6 patients had minor side effects like burning, pain and swelling at the injection site. In the Group A, those treated with oral ferrous fumarate, 14 patients had side effects out of which 8 had nausea and vomiting, 4 patients had gastritis and 2 patients had loose motion. Out of these 14 patients 8 patients took their tablets irregularly otherwise, the compliance was excellent.

In this study it was observed that parenterally administered iron sucrose elevates hemoglobin and restores iron stores better than oral ferrous fumarate during the treatment of iron deficiency anemia in pregnancy. The mean changes in hemoglobin and ferritin levels throughout the treatment were significantly higher in the intravenously administered iron group than in the orally administered iron group.

Oral iron is effective, safe, low cost, but there may be failure in the effectiveness due to non compliance, achlorhydria, inflammatory bowel diseases, or unrecognized bleeding. Non compliance is largely related to side effects. 10 to 40% of patients [10] suffer adverse gastrointestinal effects - constipation, diarrhoea, epigastric discomfort, nausea, severe abdominal pain and vomiting. They can be decreased by food, but food decreases absorption by 10 to 40%.

Iron dextran compounds are stable, strong complexes of relatively high molecular weight, long half life and relatively slow release. Life threatening anaphylactic reactions (sudden cardiovascular collapse, respiratory failure) occurred in 0.1 to 2% of patients treated with this product. 30% of patients suffered from adverse effects which include fever, arthritis, urticaria. It is associated with arthritis flare up hence contraindicated in rheumatoid arthritis. Iron sucrose complex seems to be safe with fewer and milder side effects even in patients with rheumatoid arthritis [11].

Intramuscular iron, iron-sorbitol citric acid complex causes metallic taste on tongue, nausea, vomiting and pain at the injection site [2]. Other parenteral iron preparations available are ferric gluconate, ferric citrate but are found to cause severe and extended liver necrosis [12, 13].

Iron sucrose belongs to the iron complexes of medium strong type (molecular mass between 30,000 and 100,000 Da). In the regulation of marrow proliferation iron delivery rate to the marrow is a major factor. The pharmacokinetic properties of iron dextran and iron sucrose are different. Iron dextran has a half-life of 3 to 4 days whereas iron sucrose has a terminal half-life of approximately 5 to 6 h and is quickly cleared from serum and thus rapidly available for erythropoiesis [9, 14]. It is shown in studies that in renal patients with severe IDA, 70-97% of the iron is used for erythropoiesis with only 4-6% elimination [15]. Hemoglobin concentration with intravenous iron sucrose is more rapidly increased than oral iron and intramuscular iron dextran [9].

ISC has small molecular weight hence anaphylaxis is very rare. Until now, only one case of possible anaphylactic reaction has been described. ISC is taken up mainly by the reticuloendothelial system and it is unlikely that it would be taken up by the parenchymal cells of liver, kidney, adrenal gland or other organs, hence, organic toxicity like pancreatic, myocardial or hepatic hemosiderosis is less likely even with iron sucrose complex overload.

In a random, prospective, open study done by Bayoumeu et al. [6] in 2002, 24 women were given intravenous iron sucrose in 6 slow I.V injections on days 1, 4, 8, 12, 15 and 21 with a maximum of 200 mg of iron each time and 23 women were given 240 mg oral ferrous sulfate. An increase in hemoglobin was observed on day 30 in both oral and I.V group (Not significant) but serum ferritin was higher in the IV group (P < 0.001). Similarly in our study also there was highly significant difference in the serum ferritin levels after 4 weeks of treatment (P < 0.0001) in comparison with oral group whereas there was no significant difference in terms of Hb rise after 4 weeks of treatment between oral and I.V group.

Al Momen et al. [11] in the year 1996 reported similar findings as in our study. They compared 52 women treated with intravenous iron sucrose 200 mg in 100 ml Normal saline daily till total dose was met and 59 women treated with 300 mg of oral iron sulfate and found that intravenous treatment resulted in higher hemoglobin levels in shorter periods compared with the oral treatment group (mean 6.9 versus 14.9 weeks). However, in their study 30% of the patients had poor compliance with oral treatment whereas only 14% in the oral group in our study took tablets irregularly, otherwise the compliance was excellent with ferrous fumarate.

I.V iron sucrose was well tolerated and not associated with any serious adverse effects in our study and was only associated with burning, pain and swelling at the injection site in 6 patients. It was reduced by thrombophobe ointment, ice pack and by injecting 5 cc of normal saline or distilled water at the end of I.V sucrose infusion. Previous larger studies that have investigated the safety profile of intravenous iron sucrose both during pregnancy and in the postpartum period support this finding [1, 16]. Perewunsnyk et al. [8] studied 500 women who received iron sucrose. Minor general adverse effect including a metallic taste, flushing of the face and burning at the injection site occurred in 0.5%, with doses up to 200 mg. The high tolerance of the drug has been partly attributed to slow release of iron from the complex and also due to low allergenicity of sucrose.

The compliance with oral treatment in our study was surprisingly good and was reinforced by verbal contact which is in contrast with compliance findings described in other studies. Gastrointestinal adverse effects are thought to be dose related [10] and occur more frequently at higher doses and are also related to type of iron formulation used.

In a study by Dede et al. [17] in 2004, 50 patients were included in the I.V iron sucrose group (200 mg in 100 ml normal saline daily till total dose was met) and 25 patients were included in oral ferrous sulfate group (300 mg tablet containing 60 mg elemental iron thrice daily). Blood samples were taken to evaluate levels of Hb, serum ferritin, serum iron, CRP (C-Reactive Protein), MCV (Mean corpuscular volume), TIBC (Total iron binding capacity) before the start of therapy and at days 7 and 28. It was shown in the study that intravenous iron therapy with an iron sucrose complex significantly increased serum ferritin levels within a short time with fewer adverse effects than oral iron therapy in women with post partum iron deficiency anemia. The results of this study were similar to our study.

The total dose of iron sucrose can be administered over a short period. This treatment will certainly help in reducing the risk of homologous blood transfusion during the peripartum period if used in time. Overall iron sucrose appears to be a treatment of choice with no serious side effects indicated in the rapid correction of anemia in pregnancy or restoring maternal iron stores.

## Conclusion

Intravenous iron Sucrose Complex (ISC) is safe and effective in the treatment of iron deficiency anemia during pregnancy. Intravenous iron sucrose is a most promising iron preparation for use in obstetrics because it is safe, effective and easy to administer.

## Abbreviations

CRP: C-Reactive Protein
Hb: Hemoglobin
I.V: Intravenous
IDA: Iron deficiency anemia
ISC: Iron sucrose complex
MCV: Mean corpuscular volume
NS: Normal saline
OPD: Out Patient Department
TIBC: Total iron binding capacity
WHO: World Health Organisation
